# Building school-based social capital through ‘We Act - Together for Health’ – a quasi-experimental study

**DOI:** 10.1186/s12889-018-6026-0

**Published:** 2018-09-26

**Authors:** Nanna W. Stjernqvist, Marianne Sabinsky, Antony Morgan, Ellen Trolle, Camilla Thyregod, Helle T. Maindal, Ane H. Bonde, Inge Tetens

**Affiliations:** 1 Division for Risk Assessment and Nutrition, National Food Institute, Technical University of Denmark, 2800 Kgs. Lyngby, Denmark; 2 Division for Diet, Disease Prevention and Toxicology, National Food Institute, Technical University of Denmark, 2800 Kgs. Lyngby, Denmark; 3 Diabetes Prevention Research, Steno Diabetes Center Copenhagen, 2820 Gentofte, Denmark; 4 Public Health, Glasgow Caledonian University in London, 40 Fashion Street, Spitalfields, London, E1 6PX UK; 5 Department of Applied Mathematics and Computer Science, Technical University of Denmark, Kgs. Lyngby, Denmark; 6 Present address: Department of Nutrition, Exercise and Sports, Vitality - Centre for Good Older Lives, University of Copenhagen, 1958 Frederiksberg C, Denmark; 7 Present address: Department of Public Health - Department of Health Services Research, Aarhus University, 8000 Aarhus, Denmark

**Keywords:** Social capital, Sense of belonging, Children, School, Health promoting school, Intervention, Quasi-experimental design, Multi-level logistic regression

## Abstract

**Background:**

Social capital has been found to be positively associated with various health and well-being outcomes amongst children. Less is known about *how* social capital may be generated and specifically in relation to children in the school setting. Drawing on the social cohesion approach and the democratic health educational methodology IVAC (Investigation – Vision – Action – Change) the aim of this study was to examine the effect of the Health Promoting School intervention ‘We Act – Together for Health’ on children’s cognitive social capital.

**Method:**

A quasi-experimental controlled pre- and post-intervention study design was conducted with 548 participants (mean age 11.7 years). Cognitive social capital was measured as: horizontal social capital (trust and support in pupils); vertical social capital (trust and support in teachers); and a sense of belonging in the school using questions derived from the Health Behaviour in School Children study. A series of multilevel ordinal logistic regression analyses was performed for each outcome to estimate the effect of the intervention.

**Result:**

The analyses showed no overall significant effect from the intervention on horizontal social capital or vertical social capital at the six-month follow-up. A negative effect was found on the sense of belonging in the school. Gender and grade appeared to be important for horizontal social capital, while grade was important for sense of belonging in the school. The results are discussed in relation to We Act’s implementation process, our conceptual framework and methodological issues and can be used to direct future research in the field.

**Conclusion:**

The study finds that child participation in health education can affect the children’s sense of belonging in the school, though without sufficient management support, this may have a negative effect. With low implementation fidelity regarding the Action and Change dimension of the intervention at both the school and class level, and with measurement issues regarding the concept of social capital, more research is needed to establish a firm conclusion on the importance of the children’s active participation as a source for cognitive social capital creation in the school setting.

**Trial registration:**

https://www.isrctn.com/ISRCTN85203017

**Electronic supplementary material:**

The online version of this article (10.1186/s12889-018-6026-0) contains supplementary material, which is available to authorized users.

## Background

An increasing number of studies point towards a positive association between social capital and various health and well-being outcomes in children. Positive outcomes have been found for well-being [1–3]; body mass index (BMI) [4]; physical activity [5–7]; and mental health and behavioural problems [8]. Social capital in the school setting has also been found to ‘buffer’ against inequality in children’s mental health [9] and decrease the likelihood of regular smoking [10–12], though more inconclusive findings have been found for other health risk behaviours such as alcohol use [11, 12]. While its importance to child health seems well substantiated, less research has been conducted on how social capital may be generated in adults [13], and specifically, in relation to children in the school setting [14]. This is surprising given that schools represent important communities seen from the children’s perspective where children with different social backgrounds meet and where social capital is likely to develop [12]. The school setting moreover provides opportunities where specific interventions can be tested and linked to outcomes.

### Social capital in relation to children’s health and well-being in the school setting

Social capital has been described as a resource that enhances the resilience and abilities of individuals and communities to maintain and sustain health and well-being by buffering against poor health and by providing social support and facilitating collective actions [15, 16]. In relation to children, evidence suggests that social capital and social support in the family and in the school context can operate as protective factors for their well-being [17, 18]. Social capital is a complex concept, which is what some argue gives its strength over other concepts [8]. Some of the debates are summarized here as way of background to the analysis in our study.

Firstly, social capital has been described as operating at an individual level (social network approach) and a collective level (social cohesion approach) [19]. These approaches should not be seen as mutually exclusive, and most of the published literature today recognises that it can operate at both levels [20]. Secondly, within the health field, social capital has been most commonly framed and utilised within the context of the work of Robert Putnam [13, 21] – the social cohesion approach. Putnam defines social capital as “features of social life – networks, norms and trust – that enable participants to act together more effectively to pursue shared objectives” [22]. However, in the context of children, Putnam has faced criticism for neglecting their own agency as a means of generating and using their own social capital [23]. As such, recent studies have drawn on the sociology of childhood to understand the implications of children’s agency in its construct and measurement [8, 24, 25]. Schaefer-McDaniel [25] emphasises children’s active agency and highlights three dimensions: ‘Social Networks and Sociability’, ‘Trust and Reciprocity’ and ‘Sense of Belonging/Place Attachment’. Where the first dimension is in line with the social network approach highlighting an individual’s ability to sustain and utilize one’s social network, the second relates to the social cohesion approach as applied in this study emphasising trust and norms of reciprocity. The third refers to the degree to which an individual feels that he/she is part of a collective community/environment where he/she is important and has influence [25]. The relevance for the third dimension has also been highlighted by other studies [2, 23]. Thirdly, the literature has deepened our understanding of the different qualities of different networks and interactions. These include horizontal social capital, which is further subdivided into bonding and bridging social capital as well as vertical social capital, which has also been described as ‘linking’ social capital [26–28]. Horizontal social capital tends to reflect ties that exist among people or groups of equals or near equals. By contrast, vertical social capital reflects ties of hierarchical or unequal individuals or groups who have different access to resources and power [26, 27]. Applied to the school setting, horizontal social capital therefore refers to the ties of children of equals such as classmates, whereas vertical social capital reflects the ties of unequal, such as children and teachers [10, 29].

Fourthly, in line with the different types of social capital, an empirical distinction has been made between cognitive and structural social capital. Structural social capital reflects an individual’s connectedness to a given community (e.g. participation in organisations), or what people ‘do’, whereas cognitive social capital reflects subjective feelings of trust, norms of reciprocity, connectedness or what people ‘feel’ [30]. Applied to the school setting, structural social capital may refer to child-school relations and participation in networks such as participation in extracurricular activities, school clubs or after-school centres. Cognitive social capital, on the other hand, relates to a child’s subjective perceptions of trust and support and the sense of belonging that arises from these interactions and networks [10, 29]. It is almost impossible for an individual study to embrace the complexities of social capital by including the range of distinctions and sophistications made in the literature. Rather, individual studies can make individual contributions to the pieces of the jigsaw.

This study takes a social cohesion approach and focuses on the cognitive component of social capital delineating between horizontal social capital (trust and support in pupils), vertical social capital (trust and support in teachers) and sense of belonging in the school [2, 25].

### Social capital generation and the health promoting school intervention ‘We Act – Together for health’

Putnam emphasises regular social interaction through formal and informal participation as main sources of social capital [31] and argues that “generally speaking, the more we connect with other people, the more we trust them, and vice versa” [32]. Social capital literature that focuses on how it can be generated often distinguishes between interactional processes involved in people interacting and the structural (or organisational) processes that are required to make the connections happen [33–35]. One framework that embraces these two distinct but related processes is the Health Promoting School (HPS) approach [36]. Children’s active participation is central to this approach, but equally important is the structural processes that focus on the school’s social and physical environments, active engagement with parents/and or community and health education that are required to facilitate this. In Europe, the approach is inspired by democratic health education, which emphasises the children’s genuine participation and health education that are based on a broad and positive concept of health [37]. To operationalise this within the school setting, Jensen [37] developed a practical health pedagogical methodology termed Investigation – Vision – Acting – Change (IVAC). Previous qualitative research already found HPS to be conducive for building social capital in the school setting [33, 34], but to our knowledge, no prior research has tested the effect of HPS and the IVAC methodology on the generation of children’s cognitive social capital.

This study explores these relationships based on the HPS intervention ‘We Act – Together for health’ (hereafter We Act) conducted in 2016. The intervention was developed for and targeted schoolchildren (grades 5–6) in the Danish school setting. The aim of the We Act intervention was to improve the dietary habits, physical activity, well-being and social capital among school children aged 10–12 years by increasing their health experiences and promoting a healthy school environment [38]. At the class level, we hypothesised that the children’s participation in We Act would facilitate horizontal social capital, vertical social capital, and a sense of belonging in the school among the children. Participation was anticipated to be facilitated through interactional processes of authentic dialogue, real-life and social activities mixing peers, children’s influence on content and process and working outside the classroom.

**Fig. 1:**
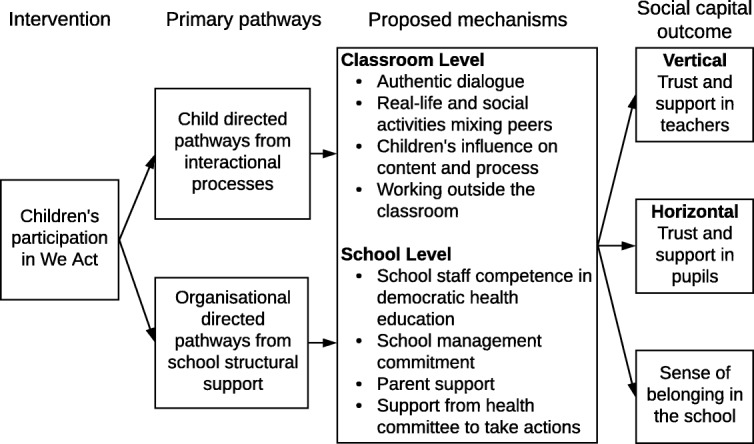
We Act intervention and proposed mechanisms for change in children’s cognitive social capital

At the school level, we hypothesised that school staff and parent participation in We Act would facilitate the same social capital outcomes among the children, which we anticipated were facilitated through organisational processes of school staff competence in democratic health education; school management commitment; parent support; and support from health committee to take actions (Fig. 1 inspired by Glass et al. [39]). The overall aim of this study was thus to investigate the effect of We Act on the children’s cognitive social capital.

## Methods

### Study design and participants

A quasi-experimental controlled pre- and post-intervention study with a three-level cluster design was applied to examine the effect of We Act on the selected social capital outcomes. The sample consisted of 656 children nested within 8 schools and 30 classes with children aged 10–12 years. The sample size was calculated in relation to the outcome dietary habits and physical activity [Personal Communication Sabinsky et al., 20 October 2017]. The schools were in both suburban and rural settings and varied in size (ranging from approximately 300 to 1200 pupils). Moreover, the schools varied with respect to the children’s socioeconomic background. Baseline data were collected using an online questionnaire between October and December 2015. Follow-up data were collected six months later in May and June 2016.

### Allocation of schools to intervention and control schools

Intervention schools and control schools were all located in Eastern Zealand in Denmark, chosen by convenience sampling. School recruitment material was sent to the municipality and/or the schools describing the intervention and the time required for participation. In total, recruitment material was sent to 27 municipalities and 210 schools. Altogether, four schools from four different municipalities signed up for the project. The four intervention schools were hereafter matched with four control schools. The control schools were selected among schools in the same municipality to make control schools as comparable as possible based on the rationale that schools within the same municipality are often exposed to the same political views and policies. Control schools were also matched on the size of the school and socioeconomic background of the families whose children attended the schools (assessed by a central person from the municipality responsible for the school area).

### Ethical issues

The study adheres to Danish ethical standards and has been approved by the Danish Data Protection Agency, 18 April 2015, ref.: 2015–41-4201. Participants were informed about the study’s objective. Teachers, children and their parents were informed that participation was voluntary, that their information would be used for research purposes only and treated confidentially, and that they could withdraw at any stage of the study. No participants withdrew from the study.

### Intervention

**Fig. 2:**
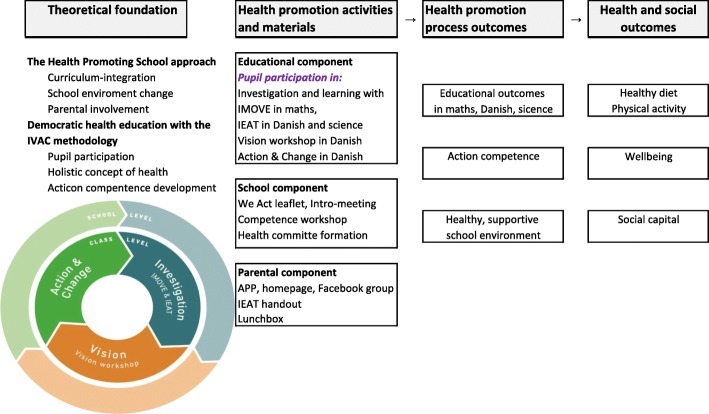
Intervention theory for We Act a HPS intervention. The intervention theory and causal assumptions for We Act were that pupils’ participation in health education following the IVAC methodology would develop their action competence in health and social competence, which, along with support from teachers, school management and parents, would initiate a change process towards a healthy supportive school environment, leading to a healthy diet, physical activity, well-being and social capital among pupils

To operationalise HPS, We Act included three components: a school component, a health educational component, and a parental component grounded on a broad and positive concept of health. All schools received the same description of the components and time frame, though with flexibility in the time frame regarding the educational component. To operationalise democratic health education within the framework of the HPS, We Act built on the IVAC methodology [37] (Fig. 2). Inspired by Paulo Freire’s [40] five-step strategy to facilitate authentic dialogue and empowerment, the IVAC methodology draws on a three-step circular pedagogical approach where pupils are actively involved in the decision-making process supported and encouraged by teachers [41]. We Act occurred at two organisational levels: the class level and at the school level. The circular process illustrates 1) the flexibility to move backwards and forwards between the different phases and 2) the process could be repeated each school year with new classes.

The school component comprised of four elements:

1. a We Act leaflet distributed before the intervention describing the objectives, the core principles, main activities including suggestions for the timetable and educational learning objectives, and the resources needed for implementation; 2) an introductory meeting with school principals, Danish language and mathematics teachers, and school nurses that aimed to prepare the conditions and agreeing upon the implementation process with the participants; 3) a competence workshop that aimed to train all participating staff; and 4) the establishment of a health committee with representatives from the school management team, teachers and health staff.

The educational component comprised of three health educational programs. These were developed to fulfil national educational objectives as well as national health educational objectives for grades 5 and 6 to avoid adding an extra burden to the schools. The programs followed the IVAC methodology. First, an Investigation phase where pupils investigate and critically discuss their physical activity with step counters (IMOVE) and their food intake and meal habits with a log book (IEAT). Second, a Vision phase (Vision Workshop) beginning with brainstorming, then voting democratically about visions for a HPS, group work based on their self-selected visions, and finally presenting their visions to an external audience outside the class (school management, parents and other classes). Hereafter, an Action & Change phase followed, where pupils work for the realisation of their visions supported by teachers, pedagogues, school management, the health committee and ideally the broader community. The complete educational component included 40 lessons over a period of 2–4 months.

The parental component comprised of five elements: 1) an application (app) for the Android and iOS platforms (HealthyKids APP), where parents can get inspiration for packed lunches; 2) a Facebook group; 3) a homepage where parents can get inspiration and exchange ideas; 4) a hand out – “My food and meals in the school” where children work on a pre-printed handout personalising it with respect to their individual preferences to be taken home and discussed with parents; and 5) lunch boxes provided to all participating pupils to increase awareness of the lunch meal at home.

### Pilot study

The different components of We Act were pilot-tested prior to implementation in collaboration with a Danish public school and a participating 5th grade class with 24 pupils and 2 teachers. Participatory observation, interviews with participating teachers and focus-group interviews with children were performed to investigate the workability of the components in the school setting in relation to the main principles of We Act. The findings suggested corrections to the procedure, assignments and time allocation for the educational process in the Investigation phase and in the Vision workshop, which were incorporated in the final version of the material. The school component was not tested in the pilot school as intended because the pilot school declined to participate in a process with a health committee in charge of an IVAC methodology at school level (parallel with IVAC at the class level). The reasons for declining were time constraints and a lack of resources. Because of this, and reluctance by other schools in the recruitment process to participate in a larger school health policy process, the school component was decreased to set-up a health committee aimed at supporting actions in the transformation from Vision to Action and Change. The parental component was tested with parents at another school.

### Evaluation study

Parallel to the implementation of We Act, a process evaluation study was conducted at the intervention schools. The purpose of this process evaluation study was to evaluate the implementation fidelity to the proposed We Act intervention components and principles, and identify the interacting context factors. Data were collected concurrently and evenly at the four schools by field visits, questionnaires, interviews and follow-up interviews during the next school term. The evaluation showed that implementation fidelity to the first phases of the educational component, the Investigation phase and Vision phase, was quite high across all four schools at the class level, though the core principal of pupil participation may not have been ‘genuine’ in the investigation phase. The implementation fidelity to the Action and Change phase at class level was on the contrary low across all four schools with a few exceptions. The implementation fidelity to the school component was low regarding the support to transition from Vision to Action and Change phase. The reach and implementation of the parental component was low across all four schools [Personal Communication, Bonde, 3 February 2018].

### Cognitive social capital

An outcome measurement was made using the WHO’s ‘Health Behaviour in School Children’ (HBSC) 2014 survey. Nine questions from the 2014 HBSC Danish contribution were selected reflecting child cognitive social capital in the school setting. These questions consisted of three latent variables representing the following three sub-indices: horizontal social capital (three items), vertical social capital (three items) and sense of belonging in the school (three items). The questions were derived reflecting both the theoretical construct and previous empirical operationalisations of child-perceived cognitive social capital in the school setting for children aged 10–12 years. The horizontal social capital index built on the work of De Clercq et al. [10]. The vertical social capital index builds on a teacher support scale derived from the HBSC international study protocol. The consistency of both scales was found through exploratory factor analysis [10, Personal Communication, Rasmussen, 29 May/2018]. Lastly, a third index of perceived cognitive social capital was constructed inspired by Schaefer-McDaniel [25] and Morgan and Haglund’s [2] emphasis on the importance of including children’s sense of belonging within the school environment. To measure the internal consistency of the indices, the coefficient of reliability - Cronbach Alpha values - were calculated for each index (Table 1). To make an easy interpretation while recognising the original ordinal response categories, the three indices were constructed as ordinal indices in line with Nielsen et al. [9] based on the number of times a respondent had answered “agree” or “strongly agree”. Hence, 1 point was given if the responder answered “agree” or “strongly agree”, and 0 points were given for negative or neutral responses. The three indices thus gave each responder 0–3 points. Hereafter, the three indices were categorised into ‘high’ =3, ‘moderate’ = 2 and ‘low’ = 1 or 0, following Nielsen et al. [9] and applied as ordinal variables as we do not know the distance between high/3, moderate/2 and low/1 and 0.

**Table 1:** Social capital items included in the analysis

Social capital items	Questions^1^	Cronbach Alpha
Cognitive		
Horizontal social capital	The students in my class enjoy being together*	0.716
	The students in my class are kind and helpful*	
	Other students accept me as I am*	
Vertical social capital	I feel that my teachers accept me as I am*	0.808
	I feel my teachers are interested in me as a person*	
	I feel a lot of trust in my teachers*	
Sense of belonging in the school	I feel I belong at this school*	0.846
	Our school is a nice place to be*	
	I feel safe at this school**	

*[strongly agree, agree, neither agree nor disagree, disagree, strongly disagree]

**[always, most of the time, sometimes, rarely, never]

^1^All questions and response categories derive from HBSC International protocols [Personal communication, Rasmussen, 28 May/2018] and has been translated into Danish following the standardised translation guidelines [57]

### Measures of covariates

Recognising the potential confounding effect, we adjusted for gender, age, migration status and for socioeconomic status (SES) at the individual level using the items from the standardised HBSC. Migration status was based on a pupil’s place of birth and their mother and father’s place of birth. This was categorised into native Danish (child born in Denmark and one or both parents born in Denmark), and non-native Danish. The latter included first-generation immigrants (both child and parents born abroad) and second-generation immigrants (child born in Denmark and parents born abroad). The child’s SES was measured by the parents’ occupational social class scheme (OSC) [42]. Pupils were asked the following questions: “Does your mother (father) have a job? If yes, write exactly what job she (he) does. Please say where she (he) works?” The children’s responses were coded by the research team in accordance with the HBSC coding recommendation. Nine categories were used for both father and mother. These categories have many similarities with the Registrar General Social Class measures [43]. Based on the highest ranking parent, each child was coded into a family social group ranging from high (I-II), medium (III-IV), low (V+ economically inactive) and unclassifiable. At the class-level, we adjusted for grade. To account for the effect of the intervention, an ‘intervention variable’ was constructed as a categorical-variable that assigned “one” to the control schools and “two” to the intervention schools.

### Statistical analysis

Multilevel ordinal logistic regression analysis was used to estimate the effect of the intervention assuming proportional odds. The hierarchical nature of the data where pupils (level 1) are nested within classes (level 2) that are nested within schools (level 3) provided the rationale for using a multi-level modelling analysis, while the ordinal nature of the outcome variables provided the rationale for the use of ordinal logistic regression analysis assuming proportional odds. However, generally it was not possible to estimate the variance component associated to variation between schools (level 3) due to a non-positive definite G matrix. We assume that this is because the amount of total variation explained by the schools is negligible compared with the variation explained by the class and individual levels. We calculated the amount of total variation at class level using the variance partition coefficient (VPC) and the latent variable method, where π^2^/3 is the variance between individuals [44]. To get a proper error structure, classes were nested with the variable intervention in the statistical models.

A series of models estimating the children’s probability (odds ratio (OR)) of reporting higher horizontal social capital, higher vertical social capital and higher sense of belonging in the school were then fitted following the bottom-up approach in line with Smiley et al. [45]. Hence, a series of models were built up step-by-step, testing for both random effects and fixed effects (covariates mentioned in Section “Measures of covariates”) using a 5% level of significance and two-sided tests. Baseline values for the respective social capital outcome were also included to adjust for potential differences at baseline. Non-significant covariates (except for the effect of the intervention) were generally removed from the models. Testing was also done for interactions between all significant covariates and the intervention. All multilevel models were fitted in SAS using the PROC GLIMMIX procedure. The Akaike Information Criterion (AIC) and the more conservative Bayesian Information Criterion (BIC) were used to compare the model fit and find the best fitting model for the data.

## Results

### Intervention participants and baseline characteristics

**Fig. 3:**
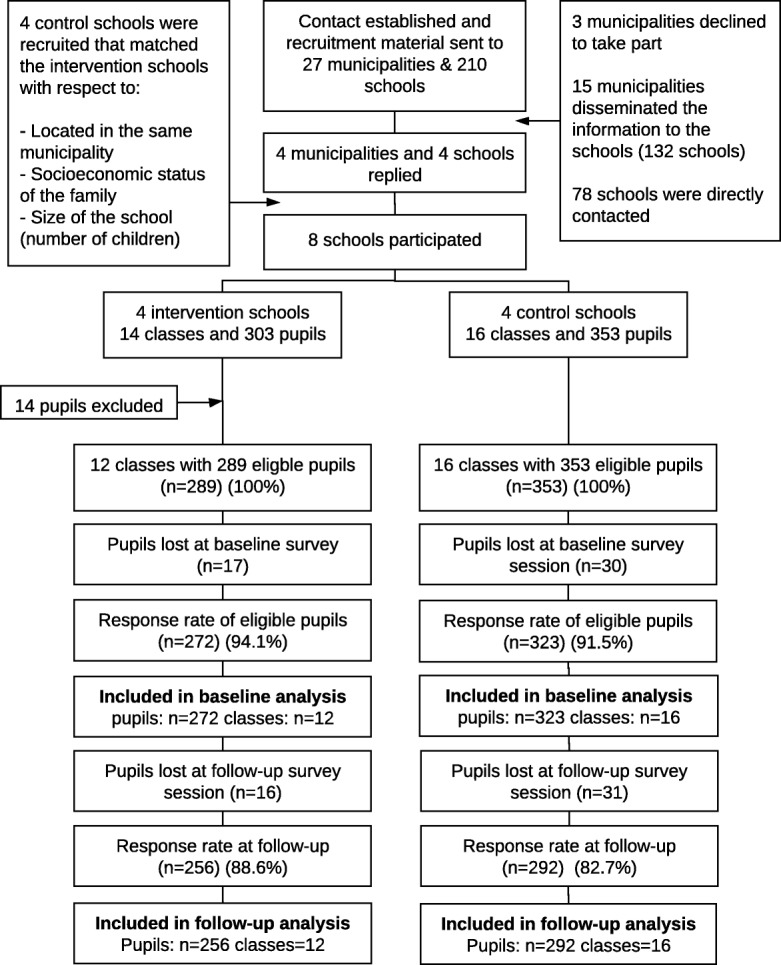
Flow diagram of recruitment and participation in We Act in Eastern Zealand, Denmark

The final baseline sample included four intervention schools with 12 classes and a total of 289 children compared with four control schools with 16 classes and 353 children. Two classes and a total of 14 children were excluded from the intervention group as these classes were special classes and not part of the target group (Fig. 3). The response rates for the baseline sample were 94.1% for intervention schools and 91.5% for control schools. The response rates for the follow-up analysis were 88.6% for intervention schools and 82.7% for control schools. The dropout analysis showed an overall consistency with respect to the included covariates and outcome measures between the group of children who only responded to the baseline and to the group of children who responded to both baseline and follow-up (Additional file 1). The children who did not participate in the follow-up analysis (absent from school, sick, or who did not want to participate) were, however, more likely to report ‘low’ and ‘moderate’ vertical social capital compared with the group of children who responded to both baseline and follow-up (*p* = 0.05) (Additional file 1). Table 2 describes the individualistic baseline characteristics of pupils at intervention and control schools showing general consistency. Children from the control schools were, however, more likely to be 6th grade and older compared with children from intervention schools (*p* = 0.002).

**Table 2:** Baseline individualistic characteristics of children by intervention and control schools

	Intervention *n* = 272	Control *n* = 323	*p*-value
Age, years [mean (SD)]	11,6 (0,68)	11,8 (0,66)	0.11^b^
Class level			0.002^a^**
5th grade	190 (70)	187 (58)	
6th grade	82 (30)	136 (42)	
Gender			0.71^a^
Boys	129 (47)	155 (48)	
Girls	143 (53)	168 (52)	
Migration status			0.51^a^
Native Danish	227 (83)	279 (86)	
Non-native Danish	45 (17)	44 (14)	
Family social group			0.89^a^
SES high	94 (34)	113 (35)	
SES medium	104 (38)	120 (37)	
SES low	37 (14)	51 (16)	
Unclassifiable	37 (14)	39 (12)	

^a^ based on chi-square test and a significant level of 0.05

^b^ based on independent t-test and a significant level of 0.05

*p ≤ 0.05; **p ≤ 0.01; ***p ≤ 0.001

### Effect of We Act on horizontal social capital

After six months, there was no significant difference between the intervention and control groups for horizontal social capital (Table 3). This is also indicated in the percentage distribution between the two groups (intervention and control) at follow-up and the effect estimate (Table 3). We did not find any significant effect of interaction between the intervention and significant level 1 and level 2 covariates on the outcome. The VPC showed that 11.3% of the individual’s horizontal social capital was attributed to class level (statistics not shown). While no significant differences were found between the intervention and control group’s perception of horizontal social capital at the six-month follow-up, boys (from both groups) were almost two times more likely to report higher horizontal social capital compared with girls (boys OR = 1.77 (1.22–2.58)). In addition, pupils (from both groups) from the 6th grade were less likely to report higher horizontal social capital at the six-month follow-up compared with pupils from the 5th grade (6th grade OR = 0.51 (0.29–0.89)) (statistics not shown). Pupils (from both groups) who responded ‘moderate’ or ‘high’ horizontal capital at baseline were furthermore significantly more likely to report higher horizontal social capital at follow-up compared with pupils who responded ‘low‘ horizontal social capital at baseline (‘high’ OR = 9.91 (6.01-16.37) and ‘moderate’ OR = 3.76 (2.18–6.48)) (statistics not shown).

**Table 3:** Effect of We Act on horizontal social capital at the six-month follow-up

	Horizontal social capital (%)				Effect (Intervention vs. Control)^a^	
	Baseline		Follow-up			
Overall *n* = 548	Intervention	Control	Intervention	Control	OR (95% CI)	*p*-value
Intervention					0.82 (0.47–1.46)	0.492
Horizontal social capital						
High	63	56	61	62		
Moderate	21	26	16	17		
Low	16	18	23	21		

*OR*, odds ratio

*p ≤ 0.05; **p ≤ 0.01; ***p ≤ 0.001

^a)^ At the individual level, the model adjusted for gender, age, migration status, baseline values for horizontal social capital and for SES. At the class level, the model adjusted for grade, while at the school level, the model adjusted for the intervention. Gender, grade and the baseline values for horizontal social capital appeared as significant covariates in the final model

### Effect of We Act on vertical social capital

At the six-month follow-up, there was no significant difference between the intervention and control groups for vertical social capital (Table 4), though the percentage distribution from baseline to follow-up for the intervention group indicates a change in a negative direction with a small effect estimate. In addition, no significant effects of interactions with the intervention and the significant level 1 and level 2 covariates were found. The VPC showed that 11.7% of the individual’s vertical social capital was attributed to classes (statistics not shown). Children (from both groups) who responded ‘moderate’ or ‘high’ vertical social capital at baseline were more likely to report higher vertical social capital at follow-up compared with children who responded ‘low‘ vertical social capital at baseline (‘high’ OR = 15.35 (8.58-27.49) and ‘moderate’ OR = 3.33 (1.76–6.29)) (statistics not shown).

**Table 4:** Effect of We Act on vertical social capital at the six-month follow-up

	Vertical social capital (%)				Effect (Intervention vs. Control)^a^	
	Baseline		Follow-up			
Overall *n* = 548	Intervention	Control	Intervention	Control	OR (95% CI)	*p*-value
Intervention					0.67 (0.37–1.22)	0.183
Vertical social capital						
High	72	71	66	69		
Moderate	16	15	13	16		
Low	11	14	21	15		

*OR* odds ratio

*p ≤ 0.05; **p ≤ 0.01; ***p ≤ 0.001

^a)^ At the individual level, the model adjusted for gender, age, migration status, baseline values for vertical social capital and for SES. At the class level, the model adjusted for grade, while at the school level, the model adjusted for the intervention. The baseline values for vertical social capital appeared as a significant covariate in the final model

### Effect of We Act on sense of belonging in the school

At the six-month follow-up, there was a significant difference between the intervention and control groups for sense of belonging in the school (Table 5). In contrast to our hypothesis, pupils from intervention schools were significantly less likely to report a higher sense of belonging in the school at follow-up compared with children at control schools with a medium effect estimate (Intervention OR = 0.54 (0.37–0.79)). This is also illustrated in the percentage distribution between the two groups at follow-up. The VPC for the classes showed that 9% of the individual’s sense of belonging in the school was attributed to classes (statistics not shown). Furthermore, children (from both groups) from the 6th grade were less likely to report a higher sense of belonging in the school at follow-up compared with pupils from the 5th grade (6th grade OR = 0.53 (0.30–0.92)) (statistics not shown). Children (from both groups) who reported a ‘high’ or ‘moderate’ sense of belonging in the school at baseline were significantly more likely to report a higher sense of belonging in the school at the follow-up compared with children who reported a ‘low’ sense of belonging in the school at baseline (‘high’ OR = 12.83 (8.09–20.34) and ‘moderate’ OR = 2.55 (1.44–4.50)) (statistics not shown).

**Table 5:** Effect of We Act on sense of belonging in the school at the six-month follow-up

	Sense of belonging in the school (%)				Effect (Intervention vs. Control)^a^	
	Baseline		Follow-up			
Overall *n* = 547	Intervention	Control	Intervention	Control	OR (95% CI)	*p*-value
Intervention					0.54 (0.37–0.79) **	0.002
Sense of belonging in the school						
High	63	67	56	65		
Moderate	13	13	13	17		
Low	23	20	31	18		

*OR* odds ratio

*p ≤ 0.05; **p ≤ 0.01; ***p ≤ 0.001

^a)^ At the individual level, the model adjusted for gender, age, migration status, baseline values for sense of belonging and for SES. At the class level, the model adjusted for grade, while at the school level, the model adjusted for the intervention. Grade and baseline values for sense of belonging in the school appeared as significant covariates in the final model

## Discussion

The current study examined the effect of the We Act intervention on the children’s cognitive social capital. No statistically significant effect of We Act on children’s probability of reporting higher horizontal social capital or higher vertical social capital was found at follow-up, though what can be considered a small negative effect estimate [46] was found on vertical social capital. Contrary to our hypothesis, a negative significant effect from We Act was found on the children’s probability of reporting a higher sense of belonging in the school at follow-up – with an effect size of medium size according to Sullivan [46].

The analysis moreover showed some differences between boys’ and girls’ horizontal social capital. The influence of gender on horizontal social capital where boys report higher trust and support in other pupils compared with girls is in line with other studies [18, 47]. The influence from grade on horizontal social capital has also been found in another study [48], which showed an interaction between grade and gender. This influence points towards a need to theoretically consider both gender and grade when working with horizontal social capital as different norms and behavioural characteristics are likely to persist between boys and girls in this age group. Moreover, grade seems to be important for the children’s sense of belonging in the school where children from the 6th grade in this study were less likely to report a higher sense of belonging in the school compared with pupils from the 5th grade at follow-up. This is in line with another study on school connectedness [49], which has to be accounted for in future studies.

To our knowledge, no prior study has tested the effect of HPS and the IVAC methodology on children’s cognitive social capital in the school setting. Therefore, it is difficult to make a comparison with other studies. An experimental study on the potential of the HPS to promote social capital showed evidence that the HPS approach is closely linked to improvement in social capital, measured by the Social Capital Index, in a primary school context. This study, however, only reported on school staff’s perceived social capital [50]. Other concepts, such as *school connectedness* or *school bonding,* are used in relation to theories such as Attachment Theory, Social Control Theory and Social Development Model [51]. With respect to the concept school connectedness, we argue that this is similar to the conceptualisation of social capital used in the current study though it does raise an issue of linguistic confusion in the literature. An experimental, comprehensive school-based intervention study designed to reduce risk and promote resilience among students (11–14 years of age) through development of a caring community showed positive effects on the items ‘sense of the school as a community’ and ‘trust and respect in teachers’ [52]. This stands in contrast to the findings of the current study, though the study by Battistich et al. [52] is limited by missing data, which made it difficult to conduct repeated measurement analysis. A similar finding appears in a Danish experimental, comprehensive school intervention study [53]. Designed to promote student (average student age 21) well-being and reduce smoking in vocational schools, Andersen et al. [53] found significant improvement in school connectedness at the 10-week follow-up in the intervention group. They also examined the effect on student support and teacher relatedness using similar scales as those applied in the current study, but did not find any effect of the intervention on these items.

It is important to consider why a negative effect on the sense of belonging in the school is found in the current study, contrary to our stated hypothesis and findings from previous studies, and to reflect on why no effect is found on the horizontal and vertical social capital. This will be discussed in relation to We Act’s implementation process; our conceptual framework and methodological issues.

Looking towards the evaluation study, the implementation fidelity to the school component was low regarding the support to the transition from Visions to Action and Change phase where the health committee was supposed to support the children’s actions. At the class level, the implementation fidelity to the Action and Change phase was also low regarding teachers supporting the children’s actions. The implementation fidelity regarding the parental component was also low. By the time of the follow-up measures, very few collective actions had occurred both at class level and school level. It is likely that the missing support from both teachers and the management level influenced the pupils who may have felt disillusioned when realising that the teachers and school management were not going to support the process further. Looking at students’ sense of community in the school, similar to the sense of belonging in the school, Vieno et al. [54] found that students’ (11–15 years of age) perception of a democratic school climate was a significant simultaneous and independent predictor of school sense of community. Thus, it is likely that the missing actions may have resulted in low perceptions of participation in rules making, which have affected the sense of belonging in the school, negatively. Similar negative consequences have been found in other studies that have worked with active involvement of pupils in school health activities in relation to pupils’ perspective [55]. The low degree of implementation fidelity does not, however, exclude flaws in our theoretical conceptualisation. It is likely that the reason for the low degree of implementation fidelity regarding supporting the children’s actions at both class and school level is due to insufficient support and guidance for this phase. Rowe and Stewart [33] highlight specific activities at school level that involve the entire school such as eating together or cross-class activities at class level as being particularly conducive for generating school social capital. In the teacher guide for the Action and Change phase, suggestions such as these were provided (i.e., preparing a dinner and being host for other classes). However, less guidance and structural support were given for the health committee at the school level and for the teachers at the class level. It is likely that the amended and decreased school-level component may have turned out to be a burden for the schools while also being too weak structurally to support the children’s genuine participation and the facilitation of collective actions based on the children’s visions, which nevertheless seem to be particularly important. A more innovative mechanism, such as the setting-up of action groups involving students and school staff (supported by an external facilitator e.g. from the municipality), may have proved more efficient as an organisational support mechanism promoting continuity with the children’s visions and active participation and ensuring intervention-retained integrity as a whole-school approach [56].

Moving on to the methodological issues, the outcome measures and the categorisation into vertical and horizontal social capital were built on the validated HBSC questionnaire and previous exploratory factor analysis as well as sense of belonging in the school. That said, there is a recognised lack of consistent measurement in social capital research, especially in relation to children, which makes comparison difficult [17]. Specifically, the selected measures also pose methodological challenges in the context of an effect study as the distribution of the answers tends to be positively skewed with lack of sensitivity, which makes positive changes difficult to detect. However, in the current study, we would rather expect a negative effect considering the effect estimates on horizontal and vertical social capital and a moderate negative effect on sense of belonging in the school. Considering the small non-statistical effect estimate on vertical social capital in a negative direction (and an even smaller non-significant negative effect on horizontal social capital), a missing effect on horizontal and vertical social capital could also be related to the power of the study. The study may be under-powered when comparing to Andersen et al. [53], although a lack of effect on both horizontal and vertical social capital was also reported in Andersen et al. [53]. While these explanations are likely to provide some insight into the unexpected findings and some comments on how to proceed in future research, a more generic problem of social capital theory is, according to Hooghe & Stolle [31], its lack of micro theoretical explanations to explain exactly which mechanisms are conducive for changes in trust and norms of reciprocity. In this study, we hypothesised social interactional and organisational processes at the school level and class level to provide one possible explanation. To advance the theory further, and avoid negative impact, additional conceptual studies are needed that can look more thoroughly into the mechanisms suggested to facilitate children’s genuine participation in different contextual school settings.

### Strengths and limitations

The We Act intervention study includes a strong theoretical framework and a robust quasi-experimental controlled pre- and post-intervention study design. It is therefore considered a strength over previous cross-sectional designs and qualitative designs, e.g., Rowe and Stewart [33, 34]. The use of multilevel logistic regression analysis within a three-level cluster design is considered advantageous as logistic regression analysis respects the categorical nature of the items. The outcome measures and the categorisation into vertical and horizontal social capital building on the validated HBSC questionnaire and previous exploratory factor analysis as well as a sense of belonging in the school is also a strength.

As two of the four intervention schools were only represented by 5th grade classes and each school is either fully in the intervention or in the control group, the effect of the variables intervention group, school and grade level are partly confounded by design, which is considered a limitation. This indicates some reservation for the conclusion regarding the estimates of the intervention. Regarding selection bias, one may consider the possibility of positive selections as intervention schools were those that answered positively to our initial contact. This seems unlikely because the decision to sign up was primarily taken by the school principal and not by the teachers who implemented most of the intervention.

## Conclusion

This study found no effect of We Act on child perceived horizontal social capital or vertical social capital. A negative effect of We Act was found on the children’s sense of belonging in the school. Child participation in health education within the framework of the HPS can thus affect the children’s sense of belonging, though without sufficient management support this may have a negative effect. Based on this study, we suggest that future studies pay more attention towards the structural and organisational level of HPS interventions. Future studies may also consider looking critically at the sensitivity of the existing measures. Our findings suggest that within the Danish school context, gender and grade appear to be important for horizontal social capital, while grade alone is important for sense of belonging in the school. This stresses the need to consider both age and gender in relation to interventions aimed at generating children’s cognitive social capital in the school.

## Additional file

Additional file 1: Dropout by groups of baseline (only) respondents compared to baseline and follow-up respondents. The table shows the statistical comparison on the individualistic characteristics and the selected outcomes between the group of children who only responded to baseline (who were lost to follow-up) and the group of children who responded to both baseline and follow-up. (PDF 21 kb)

## Abbreviations

AIC: Akaike Information Criterion
BIC: Bayesian Information Criterion
BMI: Body Mass Index
HBSC: Health Behaviour in School Children
HPS: Health Promoting School
IVAC: Investigation – Vision – Action – Change
OR: Odds Ratio
OSC: Occupational Social Class scheme
SES: Socioeconomic status
VPC: Variance Partition Coefficient
We Act: We Act – Together for health
WHO: World Health Organisation
