# Supplementary material for: Building school-based social capital through ‘We Act - Together for Health’ – a quasi-experimental study
Source: BMC Public Health. 2018 Sep 26;18:1141. doi: 10.1186/s12889-018-6026-0 (PMC6158853; doi:10.1186/s12889-018-6026-0)
Supplement: Supplementary file 1 — Dropout by groups of baseline (only) respondents compared to baseline and follow-up respondents. The table shows the statistical comparison on the individualistic characteristics and the selected outcomes between the group of children who only responded to baseline (who were lost to follow-up) and the group of children who responded to both baseline and follow-up. (PDF 21 kb) [file 12889_2018_6026_MOESM1_ESM.pdf]

Additional File 1: Dropout by groups of baseline (only) respondents compared to baseline and follow-up respondents

**Characteristics**	**Group 1: Baseline (only)**	**Group 2: Baseline and follow-up**	**p-value**[a]
Class (5th/6th) (% 5th grade)	31/16 (66%) n=47	345/203 (63%) n=548	0.68
Gender (boys/girls) (% girls)	25/22 (53%) n=47	260/288 (53%) n=548	0.45
Migration status (Native Dane/Migrant Dane) (%Native Dane)	37/10 (79%) n=47	468/80 (85%) n=548	0.22
Socioeconomic status (high, middle, low, unclassifiable) (% high)	14/18/10/5 (30%) n=47	192/205/78/73 (35%) n=548	0.21
Baseline horizontal social capital (low, moderate, high) (% high)	7/14/26 (55%) n=47	93/130/325 (59%) n=548	0.64
Baseline vertical social capital (low, moderate, high) (% high)	12/6/29 (62%) n=47	70/87/391 (71%) n=548	0.05*
Baseline sense of belonging (low, moderate, high) (% high)	7/10/30 (64%) n=47	119/71/357 (65%) n=547	0.21

[a] based on chi-square test, * p≤ 0.05; ** p≤ 0.01 ***; p≤ 0.001
